# Tau Gene Deletion Does Not Influence Axonal Regeneration and Retinal Neuron Survival in the Injured Mouse Visual System

**DOI:** 10.3390/ijms21114100

**Published:** 2020-06-08

**Authors:** Léa Rodriguez, Sandrine Joly, Julius Baya Mdzomba, Vincent Pernet

**Affiliations:** CUO-Recherche, Département d’ophtalmologie, Faculté de médecine, Université Laval and Centre de recherche du CHU de Québec-Université Laval, Quebec, QC G1V 0A6, Canada; lea.rodriguez.1@ulaval.ca (L.R.); Sandrine.Joly@crchudequebec.ulaval.ca (S.J.); julius.mdzomba.1@ulaval.ca (J.B.M.)

**Keywords:** Tau, retina, optic nerve crush, retinal ganglion cell survival, axonal regeneration, CNTF

## Abstract

In the present study, we hypothesized that the microtubule-associated protein Tau may influence retinal neuron survival and axonal regeneration after optic nerve injury. To test this hypothesis, the density of retinal ganglion cells was evaluated by immunostaining retinal flat-mounts for RNA-binding protein with multiple splicing (RBPMS) two weeks after optic nerve micro-crush lesion in Tau-deprived (Tau knock-out (KO)) and wild-type (WT) mice. Axon growth was determined on longitudinal sections of optic nerves after anterograde tracing. Our results showed that the number of surviving retinal ganglion cells and growing axons did not significantly vary between WT and Tau KO animals. Moreover, sustained activation of the neuronal growth program with ciliary neurotrophic factor (CNTF) resulted in a similar increase in surviving neurons and in growing axons in WT and Tau KO mice. Taken together, our data suggest that Tau does not influence axonal regeneration or neuronal survival.

## 1. Introduction

Traumatic injuries in the adult central nervous system (CNS) are often associated with permanent neurological impairments. This is mainly due to the limited capacity of adult axons to regenerate and to the irreversible loss of neuronal cells [1,2]. The model of rodent optic nerve injury has extensively been used to study the mechanisms responsible for the failure of axonal regeneration in the CNS. Within the first hours following optic nerve injury, the tips of severed axons form characteristic bulb-shaped terminals on the proximal side of the lesion site [3] while other cut axons withdraw [4]. At the molecular level, cytoskeleton destabilization mediates axonal collapse and prevents axonal elongation across the site of injury [2]. The disassembly and instability of neuronal cytoskeleton is thought to occur in response to inhibitory molecules present in the environment of the injured axons. In particular, myelin-associated inhibitors such as Nogo-A, Myelin-Associated Glycoprotein (MAG) and Oligodendrocyte Myelin glycoprotein (OMgp) are potent factors blocking the actin polymerization that is required in the growth cone for axonal elongation [1,2,5]. Consequently, neutralizing Nogo-A/MAG/OMgp-induced RhoA-ROCK signaling was demonstrated to be efficient at enhancing axonal growth in the injured optic nerve [6,7,8]. In addition, microtubule polymerization is sufficient to enable axonal extension from the lesion site. Indeed, topic application of the microtubule-stabilizing agent Paclitaxel (Taxol) on the injured optic nerve increased axonal growth and facilitated inflammation-induced axonal regeneration [9,10]. Similarly, Taxol was found to increase axonal extension after corticospinal tract injury [11]. Modulators of microtubule polymerization and depolymerization have been identified in the context of optic nerve axon injury. In particular, it has been proposed that microtubule-associated protein 1 B (MAP1B) and collapsin response mediator protein-2 (CRMP2) activity could be modulated to stimulate axonal regeneration in the optic nerve [12]. The development of treatments controlling microtubule polymerization and stabilization may thus allow improvement of axonal regeneration in the CNS.

Other molecules are likely to influence microtubule dynamics such as stathmins [13] and Tau. Tau is a microtubule-associated protein (MAP) especially abundant in the axons of CNS neurons where it is thought to participate in microtubule stabilization and distribution [14,15]. The propensity of Tau to aggregate in tauopathies such as fronto-temporal dementia and Alzheimer’s disease has been the focus of many studies [16,17,18]. Beside the important function attributed to Tau in neurodegenerative diseases, recent studies suggested its involvement in neuronal cell response to axonal lesion. The role of Tau has been investigated in the neuronal cell death of retinal ganglion cells in mouse models of Alzheimer’s disease and glaucoma. In a genetic mouse model of Alzheimer’s disease (3xTg), accumulation of Tau in retinal ganglion cell somata has been observed prior to cell death [19]. Interestingly, siRNA-mediated Tau silencing improved the anterograde transport that was affected by Alzheimer’s disease in retinal ganglion cell axons. Similar somatodendritic increase of Tau content has been observed in the retinal ganglion cells of glaucomatous mice [20]. In this case, intravitreal administration of Tau-targeting siRNA reduced ocular hypertension-induced retinal ganglion cell death and optic nerve axon degeneration. Although a role for Tau has been established in retinal ganglion cell death, its influence in axonal regeneration remains unclear.

To clarify this in the present study, we examined the effect of Tau deletion on retinal ganglion cell axon regeneration after mouse optic nerve crush with or without ciliary neurotrophic factor (CNTF) stimulation. Indeed, adult retinal ganglion cells have a weak intrinsic ability to grow following axonal damage. However, CNTF is a potent growth-promoting cytokine associated with inflammation [21]. Sustained adeno-associated virus (AAV)-mediated release of CNTF in the retina has been shown to produce long-distance axonal regrowth in the mouse optic nerve [22,23]. In general, treatments acting selectively on actin filament or microtubule polymerization have a relatively modest effect on axonal growth in comparison with intrinsic growth modulators such as mammalian target of rapamycin (mTOR), signal transducer and activator of transcription 3 (Stat3), kruppel-like factor 4 (KLF4) [24,25,26,27,28]. However, the combination of treatments stabilizing neuronal cytoskeleton and activating neuronal intrinsic growth mechanisms exert synergistic effects on axonal regeneration [7,9,26,29,30]. Based on this, it has been proposed that combinatorial strategies may be needed to achieve complete regeneration of injured optic nerve axons, from the back of the eye to brain targets [31,32]. Whether full axonal regeneration can be obtained using the most efficient treatments currently available is uncertain. Regenerative treatments may thus be developed with the identification of new neuronal growth mechanisms.

In the present study, we hypothesized that Tau may influence retinal neuron survival and axonal regeneration after optic nerve injury. To test this, optic nerve lesions were carried out in adult wild-type (WT) and Tau knock-out (Tau KO) mice. Our results showed that the number of surviving retinal ganglion cells and that of growing axons did not significantly vary between WT and Tau KO animals. Moreover, CNTF stimulation resulted in a similar increase in surviving neurons and growing axons with or without Tau expression. Taken together, our data suggest that Tau does not influence spontaneous and CNTF-induced axonal regeneration or neuronal survival.

## 2. Results

### 2.1. Optic Nerve Injury Decreases the Level of Tau Protein in the Retina

**Figure 1:**
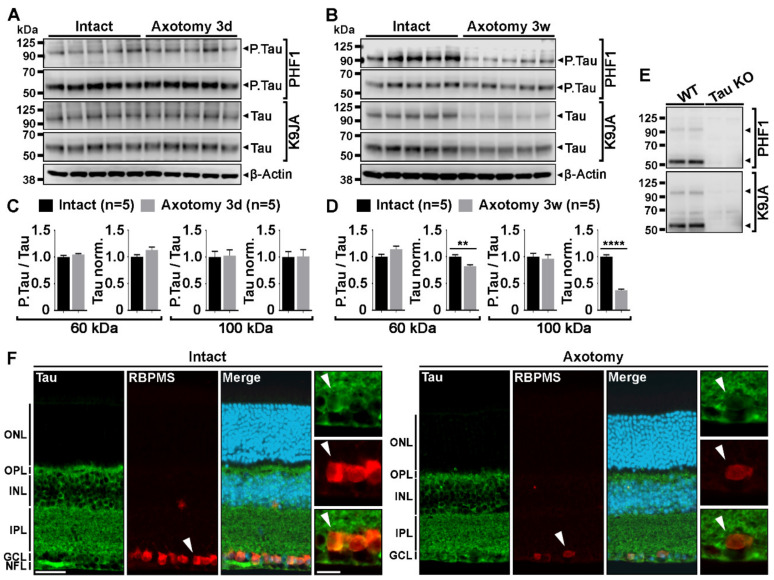
Tau protein down-regulation is associated with retinal ganglion cell death after optic nerve lesion. (**A**,**B**). Western blot analysis of phospho-Tau (P.Ser396 and P.Ser404) and Tau protein expressions in whole mouse retinal lysates at 3 days (3 d) and 3 weeks (3 w) after optic nerve injury (*n* = 5 mice per group). Injuries were carried out in the left eye. The right intact eye was used as control. Phospho-Tau and Tau expression were detected with PHF1 and K9JA antibodies, respectively. β-actin was used as loading control. (**C**,**D**). Quantitative analysis of injury-induced changes in Tau and phospho-Tau expression. Phospho-Tau expression was normalized to total Tau expression. Tau expression was normalized to β-Actin levels. Phospho-Tau and Tau expressions were normalized to the intact values (right eye). Densitometry analysis was performed for the bands at ~60 and ~100 kDa. The quantification showed no difference in phospho-Tau and Tau expression in retina at 3 days after optic nerve crush. However, after 3 weeks, Tau expression was significantly decreased. (**E**). Western blot analysis of phospho-Tau and Tau antibody specificity in Tau KO retinal lysates. (**F**). Immunofluorescent localization of injury-induced Tau protein down-regulation in RBPMS-labeled RGCs. Abbreviations: ONL = Outer Nuclear Layer, OPL = Outer Plexiform Layer, INL = Inner Nuclear Layer, IPL = Inner Plexiform Layer, GCL = Ganglion Cell Layer, NFL = Nerve Fiber Layer. Scale bars: 50 µm, close-up: 20 µm. Data are expressed as mean ± S.E.M. Statistics: Student’s t-test, **: *p* < 0.01, ****: *p* < 0.0001.

In the mouse retina, Tau is highly expressed in inner cell layers, including those of the retinal ganglion cells (RGCs) and their axons forming the nerve fiber layer [1,2]. We wondered if Tau expression changed in RGCs after optic nerve axon injury. Therefore, we examined Tau expression and its phosphorylation changes by Western blotting in whole retinal lysates, 3 days and 3 weeks after optic nerve injury (Figure 1). At 3 days, all injured RGCs are still alive although the vast majority of them are lost at 3 weeks [3,4]. PHF1 antibody was used to evaluate Tau phosphorylation at serine 396 and 404. These residues are hyperphosphorylated in pathological conditions such as in Alzheimer’s disease or glaucoma [2,5,6]. K9JA antibody was used to detect total Tau expression [7]. Using PHF1 and K9JA, two predominant forms of phospho-Tau and Tau were detected at ~60 kDa and ~100 kDa (Figure 1A,B) as previously reported by Chiasseu and colleagues [2]. The 100-kDa protein likely corresponds to big Tau which is only expressed in the retina and in neurons of the peripheral nervous system [8]. Quantitatively, densitometric analysis did not reveal a change in the level of phospho-Tau and Tau, 3 days after injury (Figure 1C). In contrast, the level of Tau expression was significantly down-regulated 3 weeks post-injury compared with intact retinae (Figure 1D). The level of phospho-Tau normalized to total Tau was however not affected (Figure 1D). The specificity of phospho-Tau and Tau antibodies was verified using Tau KO retinae as negative controls (Figure 1E). Injury-induced Tau expression changes were examined by immunofluorescence in RGCs labeled with *RNA-binding protein with multiple splicing* (RBPMS) (Figure 1F). Axotomized retinae showed weaker Tau signal in RBPMS-positive RGCs, 2 weeks after lesion, than intact retinae (Figure 1F, white arrows). These results suggest that optic nerve injury results in a massive loss of Tau attributable to RGC axon degeneration and cell death.

### 2.2. Tau Gene Deletion Does Not Influence Neuronal Survival and Axonal Growth after Optic Nerve Injury

**Figure 2:**
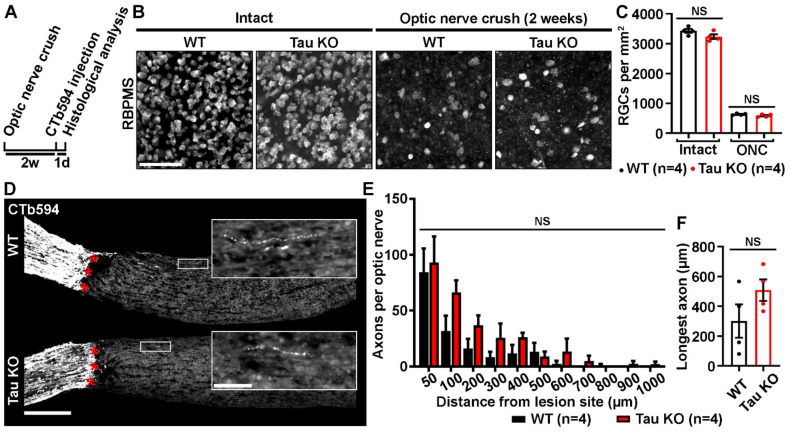
Tau deletion does not influence retinal survival and axonal growth. (**A**). Experimental timeline. Optic nerve crush (ONC) was performed on the left eye of WT and Tau KO mice. Two weeks later, axons were traced with CTb594 the day before animal sacrifice. (**B**). Influence of Tau expression on the survival of retinal ganglion cells (RGCs) 2 weeks after ONC in WT and Tau KO mice. Flat-mounted retinae were immunostained for *RNA-Binding Protein with Multiple Splicing* (RBPMS) to determine RGC density. Scale bar: 50 µm. (**C**). Quantitative analysis of RGC survival did not show difference between WT and Tau KO mice (*n* = 4 mice per group). Data are expressed as mean/mm^2^ ± S.E.M. Statistics: Student’s t-test. NS: Non-significant. (**D**). Influence of Tau deletion on axonal outgrowth at 2 weeks ONC. The growth of injured RGC axons was examined after the lesion site (red stars) in optic nerve cryosections of WT and Tau KO mice. Close-ups showed axonal sprouts beyond the injury site. Scale bars: 250 µm, close-up: 50 µm. (**E**). Quantitative analysis of axonal outgrowth. The number of axons per optic nerve was estimated in injured WT and Tau KO animals at distances ranging from 50 to 1000 μm past the injury site (*n* = 4 mice per group). Despite a trend toward an increase, Tau KO optic nerves did not display more growing axons than those of WT mice. Data are expressed as mean ± S.E.M. Statistics: Student’s t-test. NS: Non-significant. (**F**). Measurement of the longest axons in injured optic nerves. The longest axons measured in lesioned optic nerves did not reveal longer maximal extensions between Tau KO and WT mice (*n* = 4 mice per group). Data are expressed as mean ± S.E.M. Statistics: Student’s t-test. NS: Non-significant.

To determine if Tau expression influenced RGC survival and axonal regrowth after injury, WT and Tau KO mice were subjected to optic nerve micro-crush lesion. Indeed, although optic nerve axon regeneration does not spontaneously occur after injury, a very small number of sprouting axons can always be observed past the injury site [9,10]. In addition, 2 weeks post-injury, 80% of RGCs die by apoptosis in the mouse retina [11,12,13,14]. In this injury paradigm, the mechanisms of neuronal cell death may involve toxic accumulation of Tau in RGC bodies, as shown in a model of hypertensive glaucoma [2]. Neuronal survival was followed by immunostaining injured and intact retinal flat-mounts for RBPMS, a specific marker of RGCs [15] (Figure 2B). Quantitative analysis showed no difference in ganglion cell numbers between intact WT and Tau KO mice (Mean RGCs/mm^2^ ± S.E.M.; WT: 3429 ± 23 cells/mm^2^ vs Tau KO: 3235 ± 102 cells/mm^2^) and between injured WT and Tau KO mice (WT: 636 ± 29 cells/mm^2^ vs Tau KO: 587 ± 15 cells/mm^2^) (Figure 2C). Axonal regeneration was assessed on longitudinal cryosections after anterograde tracing of optic nerve axons with cholera toxin beta subunit coupled to Alexa 594 (CTb594) (Figure 2D). Few growing axons were visible after the lesion site (red stars) of WT and Tau KO optic nerves (Figure 2D). The number of axonal fibers was evaluated between 50 µm and 1000 µm past the lesion site (Figure 2E). Quantitatively, the number of CTb594-positive axons did not differ between WT and Tau KO mice. The analysis of the longest axons did not vary between Tau KO and WT optic nerves (Mean ± S.E.M.; WT: 301.2 ± 111.6 µm vs Tau KO: 508.3 ± 72.1 µm) (Figure 2F). Taken together, our observations suggest that Tau deletion does not affect the survival and axonal growth of RGCs after optic nerve injury.

### 2.3. Analysis of Tau Expression in Retinal Ganglion Cells during CNTF-Induced Survival and Regeneration

**Figure 3:**
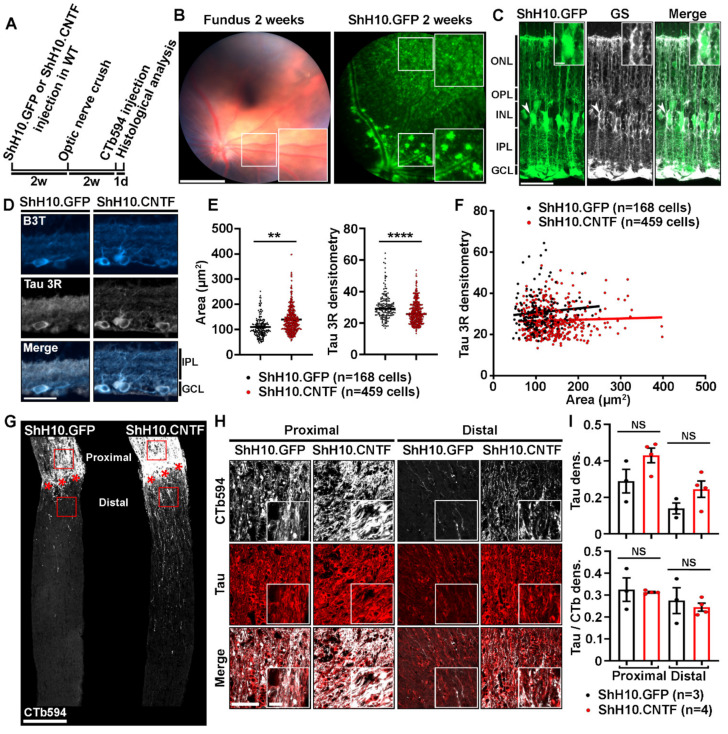
Tau expression is unchanged in the optic nerve axons of mice injected with ShH10.CNTF. (**A**). Experimental timeline. AAV2.ShH10.CNTF was used to activate injured retinal ganglion cell axon growth. AAV2.ShH10.GFP was used as control virus. Viruses were injected in the left eye of mice 2 weeks before optic nerve crush. At 2 weeks after injury, axons were traced with CTb594 the day preceding mouse sacrifice and retina and optic nerve processing. (**B**). In vivo observation of ShH10.GFP-mediated transgene expression. Retinal fundus examination with the Phoenix Micron IV microscope in anesthetized mice allowed to visualize GFP reporter expression 2 weeks after intravitreal delivery. Please note that Müller cell end feet were well visible from the periphery to the optic nerve head and all-around blood vessels. Scale bars: 500 µm, close-up: 300 µm. (**C**). Histological analysis of GFP transgene expression on retinal cryosections. Immunofluorescent staining revealed high virus-mediated expression of GFP protein in GS-expressing Müller glia at 4 weeks after ShH10 injection. Abbreviations: GS = Glutamine Synthase. GCL = Ganglion Cell Layer, IPL = Inner Plexiform Layer, INL = Inner Nuclear Layer, OPL = Outer Plexiform Layer, ONL = Outer Nuclear Layer. Scale bars: 50 µm, close-up: 10 µm. (**D**). Immunofluorescent staining of Tau 3R protein in retinal ganglion cells after infection with ShH10.GFP or ShH10.CNTF and optic nerve crush (*n* = 4 mice per group). Retinal sections were collected at 4 weeks after ShH10 injection and at 2 weeks post-injury. Abbreviations: B3T = β3-Tubulin, GCL = Ganglion Cell Layer, IPL = Inner Plexiform Layer. Scale bar: 50 µm. (**E**). Tau 3R immunofluorescence quantification in injured retinal ganglion cells. The level of Tau 3R isoform expression was determined in retinal ganglion cells by densitometric analysis after ShH10.GFP (*n* = 168 cells) and ShH10.CNTF (*n* = 459 cells) injection. Quantitative analysis showed lower Tau 3R expression in injured ganglion cell after ShH10.CNTF injection compared to ShH10.GFP injection. RGC area was determine for the same cells analyzed. Quantitative analysis showed larger injured ganglion cell area after ShH10.CNTF compared to ShH10.GFP injection. Data are expressed as mean ± S.E.M. Statistics: Student’s t-test, **: *p* < 0.01; ****: *p* < 0.0001. (**F**). Correlation analysis allowed to determine that no association could be established between retinal ganglion cell body size and the level of Tau 3R expression. Statistics: Pearson correlation analysis. NS: Non-significant. (**G**). Growing axons were traced by intravitreally injecting CTb594 in injured mice at 1 day prior to histological analysis. Axons were visualized on longitudinal sections of optic nerves two weeks after injury (red stars). Many more CTb594-labeled axons were preserved on the proximal side of the optic nerve, upstream of the lesion site, in mice treated with ShH10.CNTF than in control mice receiving ShH10.GFP. In addition, ShH10.CNTF induced robust axonal regeneration past the lesion site on the distal side of the optic nerve. Scale bar: 250 µm. (**H**). Immunofluorescent staining of Tau protein in the injured optic nerve of mice treated with ShH10.GFP or ShH10.CNTF viruses (*n* = 3/4 mice per group). Representative images showing Tau expression in axons on the proximal side of the optic nerve, i.e., upstream of the lesion site, and on the distal side at 100 µm past the lesion site. The fluorescent signal for Tau 3R was brighter with ShH10.CNTF than with ShH10.GFP vector. Scale bars: 50 µm, close-up: 10 µm. (**I**). Tau immunofluorescence quantification. Densitometric analysis revealed that Tau immunofluorescence tended to be increased in proximal and distal areas of the optic nerve of mice whose retinae were infected with ShH10.CNTF compared with control mice injected with ShH10.GFP. No difference could be observed when Tau signal was normalized to CTb594. Data are expressed as mean ± S.E.M. Statistics: Student’s t-test. NS: Non-significant.

CNTF is a potent activator of RGC survival and axonal growth in the injured optic nerve [12,16,17,18,19,20,21]. We then sought to determine if CNTF-induced axonal regeneration and neuronal survival were associated with Tau expression changes. The level of Tau was observed in RGC bodies and optic nerve axons by immunofluorescence 2 weeks after optic nerve injury in WT mice (Figure 3). To allow sustained and robust stimulation of RGCs by CNTF in the mouse retina, Müller glia was infected with ShH10.CNTF, an AAV variant containing the cDNA of *Cntf*, as in our previous studies [12,22]. AAV2.ShH10.CNTF was intravitreously injected in the left eye of WT mice 2 weeks before optic nerve crush. AAV2.ShH10.GFP was used as control vector (Figure 3A). Under fluorescence, eye fundus examination with the Micron IV imaging microscope allowed to observe GFP in retinal cells 2 weeks after intravitreal injection of ShH10.GFP. Müller glial cell end feet lining blood vessels showed strong GFP signal as previously reported (Figure 3B) [23]. On cryosections of retinae infected with ShH10.GFP, GFP was localized in glutamine synthase (GS)-containing Müller cell somata (Figure 3C, close-up) and extensions spanning the whole retinal thickness (Figure 3C). These results are consistent with previous studies showing that ShH10 viruses are powerful vectors allowing preferential transgene expression in retinal glia [12]. We chose to examine the expression of the Tau 3R isoform in injured RGCs stimulated with ShH10.CNTF. Indeed, Tau 3R has been associated with neuronal plasticity in other CNS structures [24,25,26]. Double immunofluorescent stainings for β3-Tubulin and Tau 3R were carried out on retinal cryosections of eyes infected with ShH10.GFP or ShH10.CNTF (Figure 3D). Densitometric analysis of the Tau 3R signal in β3-Tubulin-positive RGC bodies revealed a decrease of Tau 3R in retina treated with ShH10.CNTF (Figure 3E). The trophic effects of ShH10.CNTF resulted in RGC soma enlargement, in a consistent manner with what has previously been reported (Figure 3E) [27]. Interestingly, the level of Tau 3R signal could not be correlated with RGC body size changes in ShH10.GFP or ShH10.CNTF-infected retinae (Figure 3F). We wondered if the weaker level of Tau 3R observed in the soma of RGCs stimulated by CNTF was due to Tau association with microtubules in regenerating axons. To evaluate this possibility, Tau was labeled in optic nerve axons traced with CTb594 (Figure 3G–I). Observations were carried out on the proximal side of the optic nerve, i.e., in CTb594-labeled axons connected to RGC bodies upstream of the injury site, and on the distal side, i.e., beyond the injury site (Figure 3G, red squares). The intensity of Tau staining tended to be stronger in the optic nerve of mice injected with ShH10.CNTF than in those receiving ShH10.GFP treatment (Figure 3H,I). However, Tau immunofluorescence was not significantly higher once normalized to CTb594 signal, suggesting that its apparent increase may reflect the increased number of axons induced by CNTF rather than expression increase in individual axons (Figure 3H,I).

### 2.4. Tau Deletion Does Not Influence CNTF-Induced Neuronal Survival and Axonal Outgrowth in the Injured Visual System

**Figure 4:**
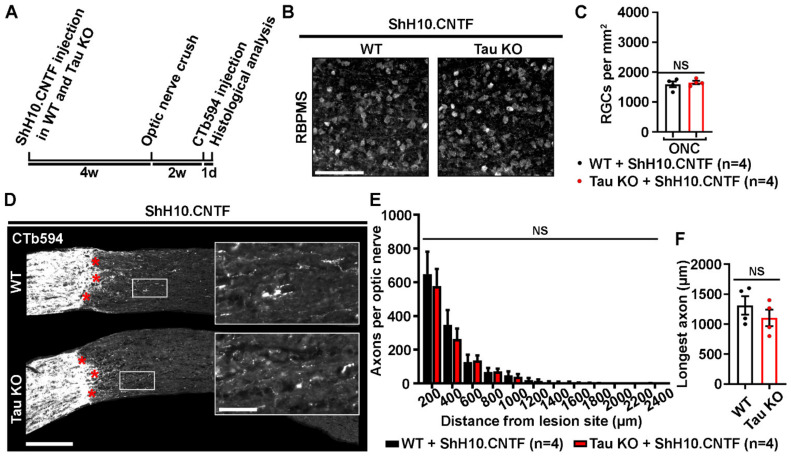
Tau deletion does not modulate CNTF-induced survival and axonal growth of injured retinal ganglion cells. (**A**). Experimental timeline. ShH10.CNTF virus was injected in the vitreous of the left eye to activate retinal ganglion cell growth mechanisms at 4 weeks before left ONC. Two weeks after injury, axons were traced with CTb594 the day before histological analysis. (**B**). Influence of Tau deletion on the survival of retinal ganglion cells (RGCs) at 2 weeks after injury. Flat-mounted retinae were immunostained for *RNA-Binding Protein with Multiple Splicing* (RBPMS) to determine RGC density in WT and Tau KO mice. Scale bar: 50 µm. (**C**). Quantitative analysis of RGC survival. The density of RGCs did not differ between WT and Tau KO mice (*n* = 4 mice per group). Data are expressed as mean/mm^2^ ± S.E.M. Statistics: Student’s t-test. NS: Non-significant. (**D**). Influence of Tau on ShH10.CNTF-induced axonal outgrowth. Axons were anterogradely traced by intravitreal injection of CTb594. The growth of injured RGC axons was examined after the lesion site (red stars) in 10-µm thick optic nerve cryosections. Close-up pictures show elongating axons beyond the injury site. Scale bars: 250 µm, close-up: 50 µm. (**E**). Quantitative analysis of axonal outgrowth. The quantification of the number of axons per optic nerve at distances ranging from 200 to 2400 μm past the lesion site did not show any difference between lesioned WT and Tau KO optic nerves (*n* = 4 mice per group). Data are expressed as mean ± S.E.M. Statistics: Student’s t-test. NS: Non-significant. (**F**). Measurement of the longest axons in injured optic nerves after CNTF-induced axonal growth. The longest axons observed on optic nerve sections after the injury site did not differ between Tau KO and WT mice. Data are expressed as mean ± S.E.M. Statistics: Student’s t-test. NS: Non-significant.

To clarify the role that Tau may have in the effects of CNTF, neuronal survival and axonal growth were compared between WT and Tau KO mice after retinal infection with ShH10.CNTF (Figure 4). Retinal and optic nerve histological analyses were realized 2 weeks after optic nerve injury (Figure 4A). The rate of surviving RGCs was estimated by immunostaining retinal flat-mounts for RBPMS (Figure 4B). The density of ganglion cells did not significantly vary between WT and Tau KO mice (Mean RGCs/mm^2^ ± S.E.M.; WT: 1589 ± 77 cells/mm^2^ vs Tau KO: 1652 ± 88 cells/mm^2^) (Figure 4C). In addition, the number of CTb594-labeled axons counted from 200 µm to 2400 µm past the lesion site was not different between WT and Tau KO optic nerves (Figure 4D,E). The length of the longest axon was similar in Tau KO optic nerves to that of WT mice (Mean ± S.E.M.; WT: 1311.2 ± 153.8 µm vs Tau KO: 1104.1 ± 138.5 µm) (Figure 4F). These results suggest that Tau is not required for the neuroprotective and regenerative effects mediated by CNTF in the injured visual system.

## 3. Discussion

The role of Tau in CNS axon regeneration is not known. Here, using the classical model of mouse optic nerve injury, we observed that the level of Tau decreased after massive RGC loss, suggesting that RGCs are an important source of Tau in the retina. Surprisingly, Tau gene deletion in KO mice did not significantly influence injured RGC death and optic nerve axonal growth. In addition, the level of Tau was not modified in regenerating axons stimulated by CNTF. CNTF-induced RGC survival and axonal regeneration did not differ in Tau KO mice from that observed in WT controls. Consequently, contrary to what has been proposed by others in vitro [28], our results suggest that Tau is not a potent modulator of neuronal survival and axonal regeneration in traumatic CNS injuries.

### 3.1. Tau Expression Changes in Injured and Regenerating RGCs

For a long time, Tau has been thought to play a major role in the growth and stabilization of CNS axons [29]. To address the role of Tau in neuronal survival and axonal regeneration, we used the classical model of optic nerve injury in adult mice. Our observations led at 3 days after injury suggest that Tau expression and phosphorylation were not modified in response to axonal lesion and prior to RGC cell death. Only a decrease in retinal Tau level was detected 3 weeks after injury, which is a time when more than 90% of RGCs are lost [3,4]. In contrast Oku and co-workers observed Tau protein up-regulation 7 days after injury in rat retinae [30]. As this time-point was not included in our study, it is possible that Tau up-regulation occurs at 7 days after optic nerve axotomy, when ~50% of RGCs are already eliminated. However, we believe that this increase in Tau level occurs too late in the process of neuronal injury response to be linked to axonal growth inhibition [31,32]. Whether the elevation of Tau protein level found by Oku et al. in RGC bodies [30] is due to gene expression or to protein miss-sorting is not clear yet. Striking Tau redistribution in the somatodendritic compartment of RGCs, at the expense of the axons, has been reported after ocular hypertension induction in glaucomatous rats [2]. Tau accumulation in RGC bodies may result from axonal transport disruption, which is recognized as an early pathological event preceding RGC death in hypertensive glaucoma [33]. After optic nerve injury as well, the slow anterograde transport of cytoskeleton proteins is interrupted in axons and is thought to restrict their regenerative capacity [34]. Consistently, the activation of axonal regeneration in peripheral nerve grafts connected to the optic nerve stump has been associated with axonal transport improvement. In the present study, we observed a reduction of Tau 3R level in the cell bodies of RGCs whose axonal growth was induced by ShH10.CNTF. This change might be indicative of a general redistribution of Tau in injured RGCs, avoiding toxic accumulation of Tau in RGC bodies and leading to its axonal relocalization to support microtubule assembly during regeneration.

### 3.2. Tau is Not Required for Optic Nerve Axon Development and Regeneration after Crush Lesion

Here, we report that Tau gene ablation does not change spontaneous and CNTF-induced axonal outgrowth. These results are surprising in regard with previous studies showing that Tau silencing strongly reduced the motility of the growth cone at the tip of growing axons in vitro [28]. More important, the number of growing axons tended to be higher in Tau KO optic nerves than in WT mice, 2 weeks after injury. This time-point is quite often used in optic nerve injury studies. However, it may not be the best moment to observe increased axonal extension. For example, inflammation-induced axonal regeneration seems optimal at 3 weeks [35]. In other studies, the examination of optic nerves at 4 weeks post-injury allowed to report sustained and robust effects of mTOR or Stat3 pathway activation on axonal growth [13,36]. In the case of Tau KO animals, the analysis of axonal growth at a later time-point than that used in our study, for example 3 weeks post-lesion, may enable the observation of significantly more regenerating axons than in control conditions. In growth-promoting conditions, despite its high level in the growing axons generated with ShH10.CNTF, Tau does not seem to be essential for axonal regrowth through the lesion site or for stabilizing the axonal shaft architecture. However, microtubule stabilization alone seems to be sufficient to promote axonal elongation in the injured spinal cord and optic nerve [10,37,38]. Indeed, the application of the microtubule-stabilizing agent Taxol on the injured optic nerve [10,38] and spinal cord [39] enhanced axonal growth. In our study, the lack of regeneration phenotype in Tau-deprived mice may result from compensatory mechanisms involving other MAPs. For example, compensatory increase of MAP1A expression has been reported in Tau KO mice at P7 but not in the mature brain [40]. The role that MAP1A may play in this case is not clear. In contrast, it has been suggested that MAP1B and Tau may have redundant functions. Overlapping roles of Tau and MAP1B have been proposed in CNS axon development based on the observation of more severe brain dysgenesis in double Tau/MAP1B KO mice than in single KO mice [28,41]. Moreover, genetic inactivation of MAP1B led to developmental abnormalities in the mouse CNS, including microphthalmia, weaker visual acuity and delayed optic nerve myelinogenesis and axonal maturation [42,43,44]. This is in contrast with Tau KO animals that did not present any phenotypic changes in the visual system. The visual function and the development of retinal projections to the brain are not affected in Tau KO mice [1,45]. In addition, detailed analysis of axonal transport in the mouse optic nerve failed to reveal alteration in mice deprived of Tau expression and in animals overexpressing Tau [46]. Altogether, these studies suggest that MAP1B may have a unique function in axonal development, contrary to Tau. This may also be true in the mechanisms of axonal regeneration. Phosphorylated MAP1B is up-regulated in injured RGCs before the beginning of apoptosis [47]. In the injured optic nerve of mice, glycogen synthase kinase 3β (GSK3β)-mediated phosphorylation of MAP1B is thought to contribute to robust axonal growth by controlling microtubule dynamics in the growth cone [9]. Although evidence suggests that MAP1B may play a role in optic nerve axon regeneration, this remains to be evaluated in MAP1B null mice. In future studies, acute silencing with interfering RNAs may help clarify the function of Tau in axonal regeneration by limiting the occurrence of potential compensatory mechanisms.

### 3.3. Tau Gene Deletion Does Not Rescue Injured RGCs from Optic Nerve Injury-Induced Cell Death

The survival of injured RGCs did not change in Tau KO mice when compared with WT animals. In the two mouse genotypes, ~20% of RGCs stayed alive 2 weeks after optic nerve injury. The rate of surviving RGCs reported in the present study is in agreement with previous studies [14]. Our data therefore suggest that Tau does not participate in injury-induced RGC apoptosis. In contrast, Tau was shown to be toxic in a hypertensive mouse glaucoma model that leads to RGC apoptosis by compressing the optic nerve head [2]. In this later study, Tau was up-regulated in RGC somatodendritic compartment. Intravitreal injection of siRNA allowed to reduce Tau expression and increased RGC survival in glaucomatous [2] and axotomized [30] animals. Based on these findings, increased RGC survival might have been expected in Tau KO retinae. The lack of survival improvement that we observed in Tau KO mice relative to other studies may be ascribed to differences in experimental design. For instance, it is important to underline that the pathological mechanisms induced by hypertensive glaucoma [2] and optic nerve lesion (present study) lead to distinct patterns of RGC loss. In the case of ocular hypertension, glaucomatous retinae undergo protracted and limited elimination of RGCs, which represents ~30% cell loss 3 weeks after glaucoma induction, compared with complete optic nerve axotomy causing ~80% of cell death after 2 weeks. The effect of Tau inactivation on RGC survival may depend on the level of cell death. One may speculate that the effects of Tau deletion on RGC survival may be visible 7 days post-optic nerve lesion, when the proportion of dead cells is limited to ~50%. The study of Oku et al., however, was carried out using complete optic nerve injury similar to us, but in rats [30]. Beside species and injury types, the experimental approach chosen to neutralize Tau function may influence the survival of RGCs. Acute siRNA-mediated silencing of Tau in the studies of Chiasseu et al. and Oku et al. may limit potential activation of compensatory mechanisms arising in Tau KO mice [2,30,40], and which may mask the effect of Tau ablation on neuronal cell death. To address this possibility in future experiments, changes in MAP expression should be analyzed in Tau KO retinae.

### 3.4. Differential Regenerative and Surviving Abilities of RGC Subtypes: Possible Implication for Tau

In our study, the survival of the whole RGC population was visualized using RBPMS as a general marker [15]. However, RGCs are composed of at least 20 different subtypes with extremely different survival and growth abilities [48]. Although the majority of RGCs die 2 weeks after optic nerve lesion and possess a very poor growth capacity, genetic approaches allowed to identify injury-resistant and regeneration-competent RGC subsets such as αRGCs and intrinsically photosensitive RGCs (ipRGCs) [49,50,51]. The proportion of αRGCs and ipRGCs accounts for only 6% and 1% of all RGCs, respectively [50,52]. In the present study, the influence of Tau deletion on the survival and regrowth of these RGC subsets was not addressed. Therefore, one cannot exclude the possibility that Tau may be involved in the survival and regenerative properties of ipRGCs or αRGCs, for example. Although this possibility is highly speculative, the non-significant increase of axonal growth that was observed in Tau KO might be attributable to a small fraction of RGCs, perhaps belonging to RGC subsets such as that of ipRGCs. To clarify this, genetic tracings of ipRGCs may be undertaken in future studies.

In summary, our results indicate that Tau is strongly expressed by RGCs in the retina. Using a KO approach, we showed that Tau is not essential for RGC survival and optic nerve axon regeneration. Further experiments will be required to examine possible compensatory mechanisms that might prevent the observation of changes in the retinal neuron response to injury.

## 4. Material and Methods

### 4.1. Animals

Adult WT and Tau KO mice (3–5 months of age) of both sexes were used for the experiments. Tau KO mice were generated by inserting the coding sequence of enhanced green fluorescent protein (EGFP) in exon 1 of *Mapt*, hence resulting into *Mapt* gene expression disruption and in the expression of a fused protein composed of EGFP and the first 31 amino acids of MAPT [53]. The founders of our mTKO mice colony (Bar Harbor, ME, USA; B6.Cg-Mapttm1 (EGFP) Klt Tg(MAPT)8cPdav/J) were from the Jackson Laboratory on C57BL/6J background. WT C57BL/6J mice were also purchased from the Jackson Laboratory (Bar Harbor, ME, USA). All animal experiments were carried out in accordance with the guidelines of the Canadian Council on Animal Care and of the Animal Welfare Committee of the *Université Laval*.

### 4.2. Optic Nerve Injuries

Optic nerve injuries were performed to study axonal regeneration in the optic nerve and neuronal survival in the retina. Mice were anesthetized with 2% isoflurane in 2% of oxygen. They were injected with buprenorphine (0.2 mg/kg) as painkiller before starting surgery. Left optic nerves were intraorbitally crushed by tying a knot with a 9-0 suture at ∼0.5 mm from the eyeball, and right eyes were used as control. Care was taken not to injure blood vessels and the integrity of the ophthalmic artery was confirmed by fundus examination just after the end of the surgery and the following day. After surgery, animals were then replaced in a clean cage at room air on a heat pad and returned to the animal accommodation room when fully alert.

### 4.3. Intravitreal Injections

Adult mice were anesthetized with isoflurane and received one dose of buprenorphine (0. 2mg/kg) before surgery. AAV.ShH10.GFP (4.45 × 10^12^ vg/mL, 2μL), AAV.ShH10.CNTF (2.01 × 10^13^ vg/mL, 2 μL) or the anterograde tracer cholera toxin *β* subunit conjugated to Alexa 594 (CTb-594, 0.5% in phosphate buffered saline (PBS), 2 μL, Thermo Fisher Scientific, MA, USA) were injected. In brief, using a 10 μL Hamilton syringe adapted with a glass tip, viral vectors, or CTb594 were intravitreously injected. The tip was inserted in the superior quadrant of the eye through the sclera into the vitreous chamber and was maintained in place for 3 min before being gently removed. A drop of surgical glue (Histoacryl^®^, Braun, Kronberg im Taunus, Germany) was applied to seal the injection site. Depending on the experiment, viral vectors were injected 2 or 4 weeks before optic nerve lesion. CTb594 was always injected the day before mouse sacrifice.

### 4.4. Fundoscopy

Two weeks after ShH10.GFP virus injection, mice were anesthetized with 2% isoflurane in 2% of oxygen. Prior to retinal examination, a drop of 1% of tropicamide (Mydriacyl, Alcon, Geneva, Switzerland) was applied on the cornea for pupil dilation. A sterile ophthalmic gel (Tear-Gel, Baush & Lomb, New York, NY, USA) was used to prevent corneal desiccation and to allow the contact between the cornea and the Phoenix Micron IV microscope objective (Phoenix Technology Group, Pleasanton, CA, USA). Retinal pictures were taken using Micron Discover V2.2.0 software (Phoenix Technology Group, Pleasanton, CA, USA).

### 4.5. Western Blot Analysis

Retinae were quickly isolated, snap frozen in liquid nitrogen and stored at −80 °C until protein lysate preparation. Retinae were homogenized for 1h on ice in Eppendorf tubes containing lysis buffer (20 mM Tris-HCl, 0.5% CHAPS, pH 8.0) and phosphatase/protease inhibitors cocktail (ThermoFisher Scientific, Rockford, IL, USA). After a 15-min centrifugation (15,000× *g* at 4 °C), supernatants were retrieved and used for protein assay (BioRad, Mississauga, ON, Canada). Retinal proteins (20 µg/well) were resolved by electrophoresis on 4–12% gradient polyacrylamide gels and transferred to nitrocellulose membranes. Nitrocellulose membranes were pre-incubated in a blocking solution of 5% BSA dissolved in TBST (0.1 M Tris-base, 0.2% Tween 20, pH 7.4) for 1 h at room temperature, incubated with primary antibodies overnight at 4 °C (Anti-Total Tau K9JA, rabbit, 1:10,000, Dako Agilent, Santa Clara, CA, USA, #A002401-2 / Anti-phospho-Tau PHF1, mouse, 1:1 000, gift from Dr Peter Davies / Anti-β-actin, mouse, 1:5000, Sigma, St Louis, MI, USA, #A5441). After washes, membranes were incubated with a horseradish peroxidase-conjugated anti-mouse or anti-rabbit antibody (1:10,000, Pierce Biotechnology, Burlington, ON, Canada). Chemiluminescent bands were detected with LiCor Western Sure Premium Chemiluminescent Substrate (Mandel, Guelph, ON, Canada) in a LiCor C-Digit blot scanner (Mandel, Lincoln, NE, USA). Band signals were quantified with the ImageJ software (Java version 1.6; NIH, Bethesda, MD, USA) and analyzed with the GraphPad Prism 7 software (San Diego, CA, USA).

### 4.6. Retinal Ganglion Cell (RGC) Survival

The survival of RGCs cell was evaluated on young and old WT and Tau KO retinal flat-mounts. Retinal flat-mounts were post-fixed overnight in 4% paraformaldehyde (PFA), rinsed with PBS three times for 45 min, incubated for 1 h in a blocking solution (PBS containing 5% bovine serum albumin (BSA), 0.3% Triton X-100, and 0.01% sodium azide) and incubated for 5 days at room temperature with anti-*RNA-binding protein with multiple splicing* antibody (Anti-RBPMS, rabbit, 1:500, PhosphoSolutions, Aurora, CO, USA, #1830-RBPMS). After intensive washing, retinal flat-mounts were incubated for 2 days at room temperature with the appropriate secondary anti-rabbit antibody diluted in blocking solution. Vectashield solution (BioLynx, Brockville, ON, Canada) was used as a mounting medium. Cells were counted in regions of 62500 µm^2^ at 0.5, 1, 1.5 and 2 mm from the optic disk in the four retinal quadrants.

### 4.7. Immunofluorescence on Retinal and Optic Nerve Cryosections

Mice were euthanized using a lethal dose of ketamine/xylazine mixture (90-10 mg/kg). Tissues were fixed by intracardial perfusion of PBS and 4% PFA solutions. Eyes and optic nerves were then immersed in a 30% sucrose solution for cryo-protection before being embedded in the Optimal Cutting Temperature (Cedarlane, Burlington, ON, Canada) medium. On Superfrost microscope glass slides, 14-µm-thick retinal or 10-µm-thick optic nerve cryosections were collected. For immunostaining, tissue slices were washed 3 times for 5 min with PBS. After washing, slides were then incubated in the blocking solution (0.5% BSA, 0.5% Triton X 100) for 1 h at room temperature and then overnight with primary antibodies at 4 °C (Anti-Total Tau K9JA, rabbit, 1:500/Anti-Tau 3R, mouse, 1:1000, EMD Millipore, Burlington, MA, USA, #05-803/Anti-β3-Tubulin, mouse, 1:1000, Promega, Madison, WI, USA, #G712A). The day after, after PBS washing, sections were incubated at room temperature with the appropriate secondary antibodies. Slides were mounted with the Vectashield mounting medium. For microscopy and image acquisition, single stack or mosaic pictures were taken with a Zeiss AxioImager M2 microscope equipped with a motorized platform and the ZEN software and an LSM 700 scanning confocal microscope (Zeiss, Oberkochen, Germany).

### 4.8. Axonal Regeneration Analysis on Optic Nerve Sections

Axons were anterogradely traced by intraocularly injecting 2 µL of 0.5% cholera toxin *β* subunit conjugated to Alexa 594 (CTb594) on day 13 post-injury (see experimental procedure in 4.3). The number of growing axons was quantified on 10-*μ*m-thick optic nerve longitudinal sections. CTb-positive axons were observed using a Zeiss AxioImager M2 microscope equipped with a motorized platform and the ZEN software at 20× magnification. The number of sprouting axons was evaluated at different distances from the lesion site. Four-six optic nerve sections were analyzed per animal. The number of axons per optic nerve (∑) was calculated using the following formula: ∑*_d_* = ∏×*R*^2^ × (average number of axons/mm)/*T*. The sum (∑) of axons at a given distance (*d*) was obtained using the average optic nerve radius (*R*) of all optic nerves after the lesion site and a thickness (*T*) of the tissue slices of 10 μm.

### 4.9. Statistical Analysis

Data were expressed as mean ± S.E.M. Statistical analysis were performed using GraphPad Prism 7 software.

## Abbreviations

MDPI	Multidisciplinary Digital Publishing Institute
DOAJ	Directory of open access journals
TLA	Three letter acronym
LD	linear dichroism
AAV	Adeno-associated virus
CRMP2	Collapsin response mediator protein-2
CNS	Central nervous system
CNTF	Ciliary neurotrophic factor
CTb594	Cholera toxin *β* subunit conjugated to Alexa 594
GSK3*β*	Glycogen synthase kinase 3 *β*
ipRGCs	intrinsically photosensitive RGCs
KLF4	Kruppel-like factor 4
MAG	Myelin-associated glycoprotein
MAP 1A/1B	Microtubule-associated protein 1A/1B
mTOR	mammalian target of rapamycin
Nogo-A	Neurite outgrowth inhibitor A
OMgp	Oligodendrocyte myelin glycoprotein
ONC	Optic nerve crush
PBS	Phosphate buffered solution
PFA	Paraformaldehyde
PHF1	Paired helical filaments 1
RBPMS	RNA-binding protein with multiple splicing
RGC	Retinal ganglion cell
siRNA	small interfering RNA
Stat3	Signal transducer and activator of transcription 3
Tau KO	Tau knock-out
WT	Wild-type
